# Efficacy of the REACH Forgiveness Intervention in Indian College Students

**DOI:** 10.3389/fpsyg.2020.00671

**Published:** 2020-04-16

**Authors:** Loren Toussaint, Everett L. Worthington, Alyssa Cheadle, Savitri Marigoudar, Shanmukh Kamble, Arndt Büssing

**Affiliations:** ^1^Department of Psychology, Luther College, Decorah, IA, United States; ^2^Department of Psychology, Virginia Commonwealth University, Richmond, VA, United States; ^3^Department of Psychology, Hope College, Holland, MI, United States; ^4^Department of Psychology, Karnatak University, Darwha, India; ^5^Quality of Life, Spirituality and Coping, Department of Medicine, Faculty of Health, Witten/Herdecke University, Herdecke, Germany

**Keywords:** REACH Forgiveness, forgiveness training, India, psychoeducation, forgiveness, well-being, wellness

## Abstract

The present study investigated the efficacy of the REACH Forgiveness psychoeducation program for the first time in Indian college students and examined theoretically-based predictors of program response based on the model of relational spirituality and forgiveness. This was an intervention experiment that spanned 5 weeks and included three measurement occasions (weeks 1, 3, 5) and two separate deliveries of the forgiveness intervention (weeks 2 and 4). Participants were *N* = 124 students at Karnatak University in Darwha, India (100 Hindu; 18 Muslim, 5 Christian, and 1 Jain). This was a manualized, secular intervention led by a trained facilitator in a group, psychoeducational format. Measures included forgiveness and unforgiveness as well as assessments of positive and negative affective states and spirituality. Participants who received immediate forgiveness training showed significant and large positive changes in forgiveness and unforgiveness, as well as, more positive affect and increased self-esteem in contrast to wait-list comparisons. Perceiving one’s offender as having a similar spirituality to oneself was a consistent predictor of response to the REACH Forgiveness program. Specifically, perceiving the offender as having a similar spirituality was related to less growth of unforgiveness and more growth in empathy, positive affect, and emotional forgiveness as a result of the psychoeducational program. The REACH Forgiveness psychoeducational approach is efficacious in an Indian college student sample, and some relational spirituality variables are important predictors of response to the program. Future studies should consider the role of Indian culture in promoting forgiveness and possibly tailor the intervention to suit the significant proportions of Hindus and Muslims in India.

## Introduction

McCullough et al. (1997) define interpersonal forgiving as “the set of motivational changes whereby one becomes (a) decreasingly motivated to retaliate against an offending relationship partner, (b) decreasingly motivated to maintain estrangement from the offender, and (c) increasingly motivated by conciliation and goodwill for the offender, despite the offender’s hurtful actions” (p. 322). These changes are often assessed by using the Transgression-Related Inventory of Motivations (McCullough et al., 1998). According to Exline et al. (2003), those motivations are closely linked with emotional forgiveness (assessed by the Emotional Forgiveness Scale, Worthington et al., 2007), which are transformations in which negative emotions toward the transgressor are replaced with positive, other-oriented emotions. Sometimes these changes involve decisions to forgive (assessed by the Decisional Forgiveness Scale, Worthington et al., 2007) which are behavioral intentions to act more benevolently toward the offender in the future.

There has been strong interest in helping people become more forgiving. Some of this interest has been due to its considerable benefits to mental and physical health, happiness, and quality of life (Toussaint et al., 2015). Numerous approaches to promoting forgiveness have been developed (Enright, 2001; Luskin, 2002; Worthington, 2008). The present study tested the REACH Forgiveness method of promoting forgiveness, which is one of the most widely-used approaches to forgiveness psychoeducation (Worthington, 2008, 2020; Worthington et al., 2000, 2018). The primary objective of this study was to examine the efficacy of the REACH Forgiveness program in India, a culture in which no program to promote forgiveness has been previously tested.

Numerous studies have examined the efficacy of the REACH Forgiveness program (for a recent review, see Worthington, 2020), and meta-analyses have borne out the efficacy of this approach (Wade et al., 2014; Akhtar and Barlow, 2018). The comprehensive meta-analysis by Wade et al. (2014) provided a couple of key findings that are relevant here. First, Wade et al. (2014) noted that levels of forgiveness were higher in individuals in forgiveness treatments compared both to individuals in alternative treatments and non-treated controls by a standardized difference of about 0.5. Second, Wade et al. (2014) characterized a dose-response relationship between the amount of time invested in the forgiveness intervention and the effect sizes on forgiveness outcomes. The relationship can be described as the following: 0.10 + 0.05^∗^(number of hours of intervention). It is important to note, however, that these findings apply to all forgiveness interventions studied and not just the REACH Forgiveness approach. Nevertheless, these meta-analytic results offer useful guidelines for using forgiveness interventions.

The REACH Forgiveness intervention is flexible and has been adapted to suit the needs of diverse groups. For instance, the REACH Forgiveness intervention has been adapted to the needs of Christians (Lampton et al., 2005; Stratton et al., 2008; Worthington et al., 2010; Greer et al., 2014). It is flexible in terms of mode of application. Self-directed learners have found the 6-hour downloadable Microsoft Word or online workbooks to be effective (Greer et al., 2014; Harper et al., 2014; Lavelock et al., 2017; Nation et al., 2018). Furthermore, the REACH Forgiveness methods have been adapted and used effectively in several cultural contexts. Participants from Australia, Ghana, Indonesia, Philippines, and international students studying in the United States have found the REACH Forgiveness method efficacious using intervention methods including 6 to 13 h in-person trainings, downloadable workbooks, and online materials (Worthington et al., 2010; Lin et al., 2014; Nation et al., 2018; Kurniati et al., 2020; Osei-Tutu et al., 2020). Specifically, culturally adapted REACH Forgiveness programs have been found to reliably decrease unforgiveness and increase forgiveness and empathy. Sometimes culturally adapting the method has strengthened it (Kurniati et al., 2020), but at other times, it has made no difference (Osei-Tutu et al., 2020).

Although the REACH Forgiveness program has been tested in the Philippines and Ghana, using the Christian-adapted version, and in Indonesia using a secular model that was adapted to the culture, REACH Forgiveness interventions have not been examined in India, the second most populous country in the world (India Population, 2019). The importance of understanding the effectiveness of forgiveness psychoeducation in a country such as India cannot be overstated. India is perhaps one of the most divided countries on earth with religion, caste, and language creating many divisions among its population (Mission India, 2017). It is imperative to better understand forgiveness in a country that has a history of intra-societal divisiveness and that represents such a large proportion of the world’s population.

The purpose of this present study was to evaluate the efficacy of the secular REACH Forgiveness program in a sample of Indians that is largely composed of Hindu and Muslim adherents. The secular version is non-sectarian. However, these religions both value forgiveness (see Rye et al., 2000). It might be that religious participants can take the secular concepts and apply their own theological practices, beliefs, and values to it—just as Rye et al. (2005) found in their *post hoc* interviews of religious participants who had been randomly assigned to either a secular or religious forgiveness intervention. Furthermore, it is important to note that previous international studies have examined unforgiveness and forgiveness as outcomes, but only one study—an internet-delivered secular version of secular REACH Forgiveness—has examined broader outcomes such as empathy, stress, and depression. It showed benefits only for empathy (Nation et al., 2018). Thus it is important to determine whether REACH Forgiveness methods are capable of generating not only benefits to forgiveness and unforgiveness, but also other closely related outcomes (e.g., involving emotional expressivity and mood and also self-esteem) that have been shown to be positively affected in United States samples (Sandage and Worthington, 2010; Sandage et al., 2015; Worthington et al., 2015; Wade et al., 2018).

Finally, Worthington and Sandage (2016) summarized numerous research studies within a model of forgiveness that considered relational spirituality. This model argues that forgiveness is dependent on relational characteristics of the victim, offender, transgression, and the sacred. There are six relationships between these four elements in the model. They include connections between victim and offender (VO), victim and transgression (VT), offender and transgression (OT), victim and sacred (VS), offender and sacred (OS), and transgression and sacred (TS). The relational characteristics all affect forgiveness. It would be difficult in a single study comprehensively to test all six relational aspects of this model. Therefore, we focused on key relational variables that we expected would be predictive in India. For example, the victim’s religious beliefs and values and dispositions are related to whatever they hold to be sacred. In addition, the victim’s perception of the offender as similarly (or differently) religious or spiritual is thought to be related to forgiveness (Davis et al., 2008) because people tend to forgive others perceived to be in their in-groups more than those perceived to be in their out-groups. Additionally, the offense could be related to the sacred if a victim considered the offense to be a desecration of something sacred. Pargament et al. (2005) have shown that such offenses are very difficult to forgive. Most research on the forgiveness and relational spirituality model has studied the forgiveness of transgressions under naturally occurring conditions. In the present article, we seek to assess person-level characteristics (i.e., religious identity as Hindu or Muslim, religious commitment, and spirituality (VS); and dispositions of gratitude, shame-proneness, and guilt-proneness), perceived spiritual similarity (OS), and desecration (TS) as predictors of responsiveness to a forgiveness training—the first study to do this.

Given the research reviewed above, we hypothesized that participants in REACH Forgiveness training, relative to a wait-list comparison group, would show decreased unforgiveness and increased forgiveness as well as benefits to overall emotions and self-esteem, and these benefits would replicate when the wait-list comparison group later received the training. In addition, we tested the predictive power of the forgiveness and relational spirituality model to predict response to the REACH Forgiveness training. Previous research has examined the predictive power of the forgiveness and relational spirituality model in predicting forgiveness in naturally-occurring settings (see Worthington and Sandage, 2016, for a comprehensive review), but none have tested the predictive power of gains in response to a REACH Forgiveness intervention. Our formally stated hypotheses are as follows:

Hypothesis 1: Receipt of forgiveness training will result in changes in forgiveness, emotional reactions, and self-esteem when the immediate training participants have completed training (Time 1 to Time 2) and when the wait-list participants have completed training (Time 2 to Time 3), and gains by the immediate training participants will be maintained (Time 2 to Time 3). Outcome measures will be three groups of measures: forgiveness and unforgiveness (i.e., unforgiveness, decision to forgive, and emotional forgiveness), emotion (i.e., empathy, negative expressivity, positive affect, negative affect), and self-esteem. This will be examined through repeated-measures analyses of variance and *t*-tests as well as independent *t*-tests.

Hypothesis 2: Variables in the relational spirituality model (Davis et al., 2008; Worthington and Sandage, 2016) will predict responsiveness to training. Those predictor variables will include person characteristics (i.e., religious identity as Hindu or Muslim, religious commitment, and spirituality; and dispositions of gratitude, shame-proneness, and guilt proneness), perception of the offender’s relationship with the sacred (Similarity of Offender to the Sacred, SOS, Scale), and the relationship of the transgression to the sacred (Sacred Desecration). Multiple regression will be used to examine correlations of each pre-training relational spirituality predictor variable with post-training outcome measures, controlling for pre-training levels of the outcome measures.

## Materials and Methods

### Design and Procedure

The present study was a REACH Forgiveness intervention in which participants were randomly assigned (by drawing a card) to immediate training or wait-list comparison groups and tested three times at baseline (T1), 2 weeks following baseline assessment (T2), and 4 weeks following baseline assessment (T3). The REACH Forgiveness training was conducted 1 week after T1 1 week before T2 for those assigned to immediate REACH training (i.e., in week 2) and 1 week after T2 but 1 week before T3 for those assigned to a wait-list comparison group (i.e., in week 4). Where “T” represents an assessment, “X” represents the REACH Forgiveness training, the design took the form:

**Table:** 

Week:	1	2	3	4	5
REACH:	T1	X	T2		T3
Control:	T1		T2	X	T3

Trainings were manualized and led by a research fellow working with one of the co-authors and trained by two of the co-authors. Training lasted 12 h and included training videos provided on www.evworthington-forgiveness.com, in-person training by one co-author, and background reading of journal articles and books on the REACH Forgiveness method. The trainings were led in group settings with group sizes of 10 to 11 members. The REACH Forgiveness program began with writing about a past offense the participant had experienced and indicating how severe and how long ago it was and completing some assessments of forgiveness (not included in study measures). During a training week of the study, 6-hour psychoeducational group interventions were completed over 3 days. The design and procedure of the study followed the best-practices for conducting psychoeducational forgiveness groups (Worthington et al., 2000).

### Participants

Participants in this study were 124 students from Karnatak University pursuing degrees in various disciplines. An overall sample size of *N* = 124 offers repeated-measures *t*-tests within the immediate treatment and wait-list groups that possess power of 0.88, assuming α = 0.05 and *d* = 0.4 [the expected repeated measures *t*-test effect size for a 6-hour training according to Wade et al. (2014)]. Independent groups *t*-tests comparing immediate treatment to wait-list participants possess power of 0.80, assuming α = 0.05 and *d* = 0.5 (the expected independent groups *t*-test effect size according to Wade et al. (2014). Participants were 55 (44%) men and 69 (56%) women. Mean and median age was 23 (*SD* = 1.44, *Range* = 21–30). Self-reported religious affiliations were Hindu (*N* = 100, 89%), Muslim (*N* = 18, 15%), Christian (*N* = 5, 4%), and Jain (*N* = 1, 0.8%). Students lived in urban (*N* = 55, 44%), rural (*N* = 25, 20%), and mixed (*N* = 44, 36%) environments and all but three (2%) students were single. Participants reported being severely hurt by the offense they experienced (*M* = 4.59, *SD* = 0.76; possible range = 1–5), which is typical of most intervention research that studies forgiveness. Although a few individuals (*N* = 7, 6%) identified offenses that occurred 36 or more months ago, resulting in a positively biased average (*M* = 12 months, *SD* = 17.10), the median time since the offense was 9 months. No differences by condition in severity or time since the offense were observed (*F*s < 3.26, *p*s > 0.07). Participants were provided with informed consent at the time they joined the intervention and again at follow-up. The study was approved by the Institutional Review Board at Karnatak University. Inclusion criteria for the study included: (a) responding “yes” to the question, “Have you experienced a hurtful incident that still bothers you enough to create negative feelings such as anger, resentment, bitterness, hate, feelings of wanting to hurt the person back, anxiety, hostility etc.?” (b) rating current unforgiveness at or above 2 (0 = *no present unforgiveness*; 1 = *a little unforgiveness*; 2 = *some substantial unforgiveness remains*; 3 = *a lot of unforgiveness*; 4 = *an extreme amount of unforgiveness*), (c) responding “yes” to the questions, “Are you ready to work on the memory of that hurtful experience in a group of other men/women with the idea of possibly forgiving the person?” and, “Are you willing to discuss the hurtful experience within the group?” There was no attrition across the 5-week study period, thus results represent an intent-to-treat design.

### REACH Forgiveness Program

The REACH Forgiveness program seeks to promote forgiveness experiences with people who wish to move through the training. Two types of forgiveness are identified: decisions to forgive and emotional forgiveness. Physical health, psychological, relational, and spiritual benefits of forgiveness are identified by group members and discussed. People are invited to make a decision to forgive. People then work through five steps of “REACH.” The term “REACH” is an acronym that represents the five key steps that interventionists can use to promote emotional forgiveness in another person. These steps include: R = Remembering the hurt, E = Empathizing with the offender, A = offering a gift of forgiveness that is Altruistic, C = Committing to forgiveness, and H = Holding on to forgiveness when doubt arises. After taking the steps to promote emotional forgiveness, people are re-invited to solidify (or make anew) a decision to forgive. Finally, generalization is sought through twelve steps to become a more forgiving person (i.e., forgivingness). Most research supporting REACH Forgiveness interventions has been secular, but a few studies have investigated Christian-accommodated REACH Forgiveness. In the current study, we utilized the secular version. The materials for the REACH Forgiveness program for groups (utilized in the present study) as well as do-it-yourself workbooks are available at: www.evworthington-forgiveness.com.

### Measures

#### Pilot Testing of Measures

Prior to conducting the experimental phase of this study, a subset of 32 participants was recruited to pilot the study measures to ensure that interpretation of the English scales was acceptable to the participants. Small portions of wording of scales were slightly modified to be more easily readable by native Kannada language readers to enhance clarity, but scales remained intact in their original English form. All scales have been shown to have acceptable psychometric properties and are scored so that higher values indicate higher levels of the construct. Coefficient alphas for all study measures are contained in Table 1.

**TABLE 1:** Coefficient alphas for all study measures.

Outcome Measures	T1	T2	T3
***Core Measures***			
Unforgiveness	0.97	0.99	0.93
Decisional Forgiveness	0.76	0.63	0.29
Emotional Forgiveness	0.87	0.98	0.91
***Emotion Measures***			
Empathy	0.98	0.99	0.96
Expressivity	0.71	0.73	0.77
Positive Affect	0.96	0.98	0.89
Negative Affect	0.95	0.99	0.92
***Self-Perception***			
Self-Esteem	0.56	0.95	0.95
***Measures in the Forgiveness and Relational Spirituality Model (Worthington and Sandage, 2016)***			
Religious Affiliation	–		
Religious Commitment	–		
Spirituality	–		
Dispositional Gratitude	0.72		
Shame-Proneness	0.95		
Guilt-Proneness	0.67		
Similarity of Offender Spirituality	0.86		
Sacred Desecration	0.95		

### Outcome Measures (T1, T2, and T3)

#### Unforgiveness

Unforgiveness was assessed using the Transgression-Related Interpersonal Motivations Inventory-18 (McCullough et al., 2010). This scale consists of 18 items with responses of 1 = *strongly disagree* to 5 = *strongly agree* scale. An example item is, “I have given up my hurt and resentment.” Scores on this measure possess good estimated internal consistency (αs > 0.85), moderate 8-week, test-retest temporal stability (*r* = 0.50), and excellent factorial and construct validity (McCullough et al., 1998, 2001, 2006).

#### Decisional Forgiveness Scale

The extent to which an individual had made a decision to forgive their offender was measured with the Decisional Forgiveness Scale (Worthington et al., 2007). This scale assessed behavioral intentions to act more forgiving toward the offender. It consists of eight items with responses of 1 = *strongly disagree* to 5 = *strongly agree* scale. An example item is, “I will try to act toward him or her in the same way I did before he or she hurt me.” Scores on this measure have good estimated internal consistency (α = 0.78), good 3-week, test-retest temporal stability (*r* = 0.73), and excellent factorial and construct validity (Worthington et al., 2007).

#### Emotional Forgiveness Scale

The degree to which an individual felt emotional forgiveness toward their offender was measured with the Emotional Forgiveness Scale (Worthington et al., 2007). This scale consists of eight items with responses of 1 = *strongly disagree* to 5 = *strongly agree* scale. An example item is, “I feel love toward him or her.” Scores on this measure have good estimated internal consistency (α = 0.81), good 3-week, test-retest temporal stability (*r* = 0.73), and excellent factorial and construct validity (Worthington et al., 2007).

#### Empathy

Batson’s Empathy Adjectives were used to measure the extent to which an individual felt emotional, other-focused feelings of empathy toward the offender (Batson et al., 1987). The scale has eight items with responses of 1 = *not at all* to 6 = *extremely* scale. Example adjectives are, “sympathetic,” “moved,” and “compassionate.” Scores on this scale have acceptable estimated internal consistency (αs ≈0.70) and robust factorial and construct validity (Batson et al., 1987; Niezink et al., 2012).

#### Negative Expressivity

The Berkeley Expressivity Scale assesses bodily (e.g., facial and postural) changes that typically reflect emotional experience such as frowning or smiling (Gross and John, 1997). The scale contains three subscales including positive and negative expressivity and a subscale that indexes impulse strength. Only the negative expressivity scale was included in the present study and is scored so that high values indicate a low level of negative expressivity. The negative expressivity subscale contains six items with responses of 1 = *strongly disagree* to 7 = *strongly agree*. An example item is, “Whenever I feel negative emotions, people can easily see exactly what I am feeling.” Scores on this measure have good estimated internal consistency (α = 0.86) and excellent factorial and construct validity (Gross and John, 1997, 2003).

#### Positive and Negative Affect

The Positive and Negative Affect Scales were used to assess positive and negative mood (Watson et al., 1988). Each scale consists of 10 items with responses of 1 = *very slightly or not at all* to 5 = *extremely scale*. Example positive and negative affect items, respectively, are, “interested,” “excited,” and “strong” and “distressed,” “upset,” and “hostile.” Scores on this measure have good estimated internal consistency (αs > 0.84), good 1-week, test-retest temporal stability (rs ≈0.80), and excellent factorial and construct validity (Watson et al., 1988).

#### Self-Esteem

The Rosenberg Self-Esteem Scale is the most widely used instrument to assess a participant’s global self-evaluation of personal worth (Rosenberg, 1965). The scale has 10 items with responses of 1 = *strongly agree* to 4 = *strongly disagree* scale. An example item is, “I take a positive attitude toward myself.” Scores on this measure have excellent estimated internal consistency (α = 0.91) and item convergence and divergence characteristics and excellent factorial and clinical validity (Sinclair et al., 2010).

### Measures Within the Forgiveness and Relational Spirituality Model (Davis et al., 2008; Worthington and Sandage, 2016; T1 Only)

#### Religiousness and Spirituality

Religiousness and spirituality were measured with three single-item measures. Religious affiliation was measured by asking, “What is your religious affiliation?” Response options were: Buddhism, Hindu, Muslim, Christian, Jain, and Sikh. Religious commitment was assessed with the item, “How committed are you to your religion?” Spirituality was assessed with the item, “How intense is your spiritual life?” Response options for religious commitment and spirituality items were 1 = *not at all* to 5 *totally*.

#### Trait Gratitude

The Gratitude Questionnaire was used to assess the tendency to recognize and respond with positive emotion to the good will and generosity of others and positive experiences in one’s life (McCullough et al., 2002). The scale has six items with responses of 1 = *strongly disagree* to 7 = *strongly agree*. An example item is, “I have so much in life to be thankful for.” Scores on this measure have good estimated internal consistency (α = 0.82) and excellent factorial and construct validity (McCullough et al., 2002).

#### Shame-Proneness and Guilt-Proneness

Shame and guilt were assessed using the Test of Self-Conscious Affect (Tangney et al., 2000). This scale contains independent subscales that measure characterological shame and the experience of guilt. Sixteen scenarios are presented to participants and each scenario contains separate shame and guilt responses. An example scenario is, “While out with a group of friends, you make fun of a friend who’s not there.” The shameful response, “You would feel small … like a ‘rat,”’ and the guilty response, “You would apologize and talk about that person’s good points” have response options of 1 = *not likely* to 5 = *very likely*. Scores on this measure have acceptable estimated internal consistency (α = 0.74 shame; α = 0.69 guilt), moderate 3- 5-week, test-retest temporal stability (*r* = 0.85 shame; *r* = 0.74 guilt), and excellent construct validity (Tangney, 1990, 1996; Fontaine et al., 2001).

#### Similarity of Offender Spirituality

The Similarity of Offender Spirituality scale was used to assess a victim’s appraisals of spiritual similarity of the offender and victim (Davis et al., 2009). Similarity in basic religious beliefs as well as humanistic similarity was assessed. The scale has nine items with response options of 0 = *completely disagree* to 6 = *completely agree* scale. An example item is, “I thought about how similar my basic religious beliefs were to his/hers.” Scores on this measure have good internal consistency (α = 0.87) and excellent factorial and construct validity (Davis et al., 2009).

#### Sacred Desecration

The extent to which a participant perceived the offense as an intentional and direct or indirect violation against God or one’s belief in a higher power or against one’s religious beliefs or spirituality or anything the participant holds sacred was assessed using the Sacred Desecration scale (Pargament et al., 2005). The scale has 10 items with responses of 1 *not at all* to 5 *very much* scale. An example item is, “This event was both an offense against me and against God.” Scores on this measure have excellent estimated internal consistency (α = 0.92) and excellent factorial and construct validity (Pargament et al., 2005).

### Analyses

To examine the first hypothesis, analyses included mixed-model analyses of variance (significance tests = *F*, effect size = η_p_^2^) and independent groups (effect size = *d*) and repeated-measures (effect size = *d*) *t*-tests. Models were estimated with and without covariates, but the pattern and significance of the results were virtually identical. Thus, results are reported without covariates included in the model. Analyses addressing the first hypothesis examined all eight outcome variables measured at T1, T2, and T3, including unforgiveness, decisional and emotional forgiveness, empathy, negative expressivity, affect, and self-esteem. To examine the second hypothesis, analyses included multiple regression models in which post-training forgiveness, emotion, and self-perception outcomes were predicted by pre-training levels of the outcomes themselves and relational spirituality predictors. All variables adhered to assumptions regarding linearity and normality, and statistical significance was set at *p* < 0.006 (Bonferroni adjustment α = 0.05/8 outcomes) to control type I error inflation that results from examining eight non-independent outcomes.

## Results

In Table 2, we report the means and standard deviations for immediate-training and wait-list comparison participants across all time points. As expected, immediate-training and wait-list comparison participants showed improvements across time, but only at appropriate times (T1-T2 for immediate-training and T2-T3 for wait-list comparisons). This is evidenced by significant condition by time interactions (see Table 3; λs ≤ 0.49, *F*s ≥ 62.58, *p*s ≤ 0.001, η_p_^2^s ≥ 0.51) that were present for all outcome variables except expressivity (λ = *0.95, F* = 2.95, *p* = 0.060, η_p_^2^ = 0.05). Repeated measures *t*-tests revealed that participants in the immediate-training condition showed significant improvements on all variables (*t*s ≥ 9.43, *p*s ≤ 0.001, *d*s ≥ 1.20) except expressivity (*t* = −2.54, *p* = 0.014, *d* = −0.32) from T1 to T2 and either maintained these changes (i.e., non-significant changes) from T2 to T3 or showed continued improvement (see Table 4; *t*s ≥ 3.15, *p*s ≤ 0.003, *d*s ≥ 0.40). Repeated measures *t*-tests revealed that participants in the wait-list comparison condition showed no changes from T1 to T2 on any outcome variables. Significant improvements (*t*s ≥ 5.04, *p*s ≤ 0.001, *d*s ≥ 0.65) occurred from T2 to T3 for wait-list participants on all outcome variables (see Table 4). For participants in both immediate-training and wait-list comparison conditions significant improvements (*t*s ≥ 3.34, *p*s ≤ 0.001, *d*s ≥ 0.42) on all outcomes occurred from T1 to T3 (see Table 4). Independent *t*-tests were used to examine differences between immediate-training and wait-list comparisons at T1, T2, and T3 and showed that at T1 there were no significant differences on any outcome variable (*t*s ≤ 2.30, *p*s ≥ 0.023, *d*s ≤ 0.21), at T2 there were significant differences on all variables favoring improvement of immediate-training over wait-list comparisons (*t*s ≥ 3.13, *p*s ≤ 0.002, *d*s ≥ 0.28) except on expressivity (*t*s = 1.58, *p* = 0.116, *d* = 0.14), and at T3 there were no significant differences by condition (*t*s ≤ 1.93, *p*s ≥ 0.055, *d*s ≤ 0.17) on any variable (see Table 5).

**TABLE 2:** Means and standard deviations for immediate training and wait-list comparison participants across time.

	Time 1		Time 2		Time 3	
	*M*	*SD*	*M*	*SD*	*M*	*SD*
***Core Measures***						

**Unforgiveness**						
Immediate Training	3.93_a_	1.13	1.48_b_	0.54	1.37_b_	0.66
Wait-List Comparison	4.15_a_	0.97	4.13_a_	1.03	1.32_b_	0.49
**Decisional Forgiveness**						
Immediate Training	2.69_a_	0.82	3.77_b_	0.32	3.94_b_	0.41
Wait-List Comparison	2.44_a_	0.71	2.53_a_	0.79	3.97_b_	0.40
**Emotional Forgiveness**						
Immediate Training	2.12_a_	0.98	4.31_b_	0.67	4.64_c_	0.67
Wait-List Comparison	1.76_a_	0.80	1.79_a_	0.91	4.59_b_	0.61

***Emotion Measures***						

**Empathy**						
Immediate Training	2.03_a_	1.42	4.67_b_	0.99	5.21_c_	0.99
Wait-List Comparison	1.81_a_	1.18	1.95_a_	1.29	5.19_b_	0.86
**Expressivity**						
Immediate Training	5.56_a_	0.85	5.81_a,b_	0.93	5.91_b_	0.88
Wait-List Comparison	5.53_a_	0.97	5.57_a_	0.76	5.91_b_	0.93
**Positive Affect**						
Immediate Training	1.94_a_	1.06	3.95_b_	0.43	4.49_c_	0.67
Wait-List Comparison	1.81_a_	1.00	1.78_a_	1.08	4.34_b_	0.61
**Negative Affect**						
Immediate Training	3.95_a_	1.12	1.32_b_	0.53	1.25_b_	0.62
Wait-List Comparison	4.15_a_	1.07	4.10_a_	1.33	1.22_b_	0.55

***Self-Perception***						

**Self Esteem**						
Immediate Training	1.70_a_	0.29	3.00_b_	0.78	3.25_c_	0.89
Wait-List Comparison	1.77_a_	0.39	1.61_b_	0.30	3.26_c_	0.83

*Means in each row that share subscripts do not differ significantly at Bonferroni-adjusted *p* < 0.006.*

**TABLE 3:** Condition by time interaction statistics for all outcome variables.

	λ	*F*	*p*	η_p_^2^
***Core Measures***				
Unforgiveness	0.27	160.41	<0.001	0.73
Decisional Forgiveness	0.49	62.58	<0.001	0.51
Emotional Forgiveness	0.35	112.76	<0.001	0.65
***Emotion Measures***				
Empathy	0.48	66.23	<0.001	0.52
Expressivity	0.95	2.95	0.060	0.05
Positive Affect	0.37	102.57	<0.001	0.63
Negative Affect	0.34	117.64	<0.001	0.66
***Self-Perception***				
Self-Esteem	0.35	113.43	<0.001	0.65

**TABLE 4:** Within-participant changes for immediate training and wait-list comparison participants.

	Time 1 vs. Time2			Time 2 vs. Time 3			Time 1 vs. Time 3		
	*t*	*p*	*d*	*t*	*p*	*d*	*t*	*p*	*d*
***Core Measures***									
**Unforgiveness**									
Immediate Training	13.09	<0.001	1.66	1.44	0.154	0.18	12.54	<0.001	1.59
Wait-List Comparison	0.34	0.736	0.04	15.53	<0.001	1.97	16.12	<0.001	2.05
**Decisional Forgiveness**									
Immediate Training	–9.43	<0.001	–1.20	–2.51	0.015	–0.32	10.69	<0.001	–1.36
Wait-List Comparison	–1.42	0.160	–0.18	–12.84	<0.001	–1.63	–13.26	<0.001	–1.68
**Emotional Forgiveness**									
Immediate Training	–12.63	<0.001	–1.60	–3.20	0.002	–0.41	–14.23	<0.001	–1.81
Wait-List Comparison	–0.53	0.596	–0.07	–16.60	<0.001	–2.11	–18.35	<0.001	–2.33

***Emotion Measures***									

**Empathy**									
Immediate Training	–11.09	<0.001	–1.41	–2.99	0.004	–0.38	–13.69	<0.001	–1.74
Wait-List Comparison	–1.16	0.251	–0.15	–15.29	<0.001	–1.94	–17.24	<0.001	–2.19
**Expressivity**									
Immediate Training	–2.54	0.014	–0.32	–1.32	0.192	–0.17	–3.34	0.001	–0.42
Wait-List Comparison	–0.32	0.752	–0.04	–5.04	<0.001	–0.65	–5.15	<0.001	–0.66
**Positive Affect**									
Immediate Training	–12.91	<0.001	–1.64	–6.47	0.000	–0.82	–12.93	<0.001	–1.64
Wait-List Comparison	0.44	0.660	0.06	–12.96	<0.001	–1.65	–14.27	<0.001	–1.81
**Negative Affect**									
Immediate Training	13.56	<0.001	1.72	0.93	0.356	0.12	13.29	<0.001	1.69
Wait-List Comparison	0.57	0.569	0.07	13.00	<0.001	1.65	15.48	<0.001	1.97

***Self-Perception***									

**Self-Esteem**									
Immediate Training	–11.25	<0.001	–1.43	–5.92	<0.001	–0.75	–11.66	<0.001	–1.48
Wait-List Comparison	2.94	0.005	0.37	–12.86	<0.001	–1.63	–12.08	<0.001	–1.53

*Repeated measures *t*-tests and effect sizes (*d*).*

**TABLE 5:** Comparison of immediate training and wait-list comparisons at all time points.

	Time 1			Time 2			Time 3		
	*t*	*p*	*d*	*t*	*p*	*d*	*t*	*p*	*d*
***Core Measures***									
Unforgiveness	−1.15	0.253	−0.10	−17.98	<0.001	−1.61	0.51	0.613	0.05
Decisional Forgiveness	1.83	0.070	0.16	11.37	<0.001	1.02	−0.47	0.640	−0.04
Emotional Forgiveness	2.30	0.023	0.21	17.48	<0.001	1.57	0.46	0.649	0.04
***Emotion Measures***									
Empathy	0.91	0.365	0.08	13.17	<0.001	1.18	0.11	0.913	0.01
Expressivity	0.13	0.895	0.01	1.58	0.116	0.14	0.00	1.000	0.00
Positive Affect	0.69	0.495	0.06	14.67	<0.001	1.32	1.31	0.192	0.12
Negative Affect	−1.01	0.315	−0.09	−15.31	<0.001	−1.38	0.34	0.738	0.03
***Self-Perception***									
Self Esteem	−0.98	0.328	−0.09	13.08	<0.001	1.17	0.09	0.925	0.01

*Independent *t*-tests and effect sizes (*d*).*

Table 6 contains the regression coefficients for all relational spirituality variables predicting all post-training forgiveness, emotion, and self-perception outcomes, controlling for pre-intervention levels of these outcomes. Controlling for pre-training levels of each construct, relational spirituality predictors accounted for 52, 8, 37, 24, 74, 43, 57, and 82% of the variance in post-intervention levels of unforgiveness, decisional forgiveness, emotional forgiveness, emotional expressivity, positive affect, negative affect, and self-esteem, respectively. With regard to unique predictors of change in outcomes, similarity of offender spirituality was the most consistent predictor showing significant, negative associations with growth in unforgiveness, emotional expressivity, and self-esteem (βs = −0.17 to −0.42, *p*s ≤ 0.005) and significant, positive associations with empathy (β = 0.40, *p* = 0.002), and positive affect (β = 0.46, *p* < 0.001) and a positive association that approached significance for emotional forgiveness (β = 0.32, *p* = 0.008). Shame-proneness showed significant, positive associations with growth in emotional forgiveness, emotional expressivity, and self-esteem (βs = 0.43 to 0.67, *p*s ≤ 0.003). Trait gratitude was negatively associated with growth in self-esteem (β = −0.15, *p* = 0.001). Guilt-proneness was negatively associated with growth in negative affect (β = −0.33, *p* < 0.001) and perceiving the offense as a sacred desecration was positively associated with growth in positive affect (β = 0.35, *p* = 0.007).

**TABLE 6:** Relational spirituality predictors of forgiveness, emotion, and self-perception outcomes resulting from REACH Forgiveness training.

				Decisional			Emotional					
	Unforgiveness			Forgiveness			Forgiveness			Empathy		
	*B*	β	*p*	*B*	β	*p*	*B*	β	*p*	*B*	β	*p*
Pre-Training	−0.35	−0.73	0.000	0.01	0.02	0.931	−0.27	−0.40	0.009	−0.08	−0.11	0.465
**Person Variable**												
Hindu	−0.12	−0.09	0.475	0.02	0.02	0.888	0.18	0.11	0.453	0.08	0.03	0.843
Muslim	−0.13	−0.09	0.482	0.12	0.11	0.516	0.19	0.10	0.471	−0.18	−0.07	0.672
Religious Commitment	0.03	0.05	0.620	−0.11	−0.25	0.085	−0.06	−0.08	0.503	−0.10	−0.09	0.486
Spirituality	−0.10	−0.17	0.108	0.07	0.16	0.265	0.09	0.11	0.351	0.05	0.05	0.716
Gratitude	0.07	0.10	0.180	−0.10	−0.19	0.065	0.13	−0.14	0.104	−0.04	−0.03	0.752
Shame-Proneness	−0.17	−0.32	0.020	0.04	0.10	0.591	0.29	0.43	0.003	0.39	0.39	0.014
Guilt-Proneness	0.19	0.12	0.124	0.20	0.18	0.096	0.19	0.10	0.266	−0.15	−0.05	0.585
**Offender’s Relationship with the Sacred**												
Similarity of Offender Spirituality	−0.21	−0.42	0.000	−0.04	−0.11	0.378	0.20	0.32	0.008	0.37	0.40	0.002
**Relationship of the Transgression to the Sacred**												
Sacred Desecration	0.06	0.13	0.341	−0.09	−0.27	0.153	−0.07	−0.12	0.412	0.17	0.19	0.221

	**Expressivity**			**Positive Affect**			**Negative Affect**			**Self-Esteem**		
	***B***	**β**	***p***	***B***	**β**	***p***	***B***	**β**	***p***	***B***	**β**	***p***

T1	0.35	0.31	0.000	−0.28	−0.54	0.000	−0.14	−0.31	0.016	0.14	0.05	0.310
**Person Variable**												
Hindu	0.10	0.04	0.635	0.18	0.13	0.343	−0.06	−0.04	0.707	0.06	0.03	0.709
Muslim	0.10	0.04	0.675	0.15	0.09	0.495	0.02	0.02	0.894	0.16	0.07	0.378
Religious Commitment	−0.13	−0.12	0.106	−0.05	−0.08	0.463	0.06	0.09	0.327	0.02	0.02	0.735
Spirituality	0.14	0.13	0.089	0.07	0.11	0.327	−0.02	−0.04	0.700	0.01	0.01	0.941
Gratitude	−0.16	−0.12	0.026	0.07	0.09	0.262	0.12	0.16	0.020	−0.17	−0.15	0.001
Shame-Proneness	0.43	0.45	0.000	0.01	0.02	0.885	−0.10	−0.17	0.178	0.57	0.67	0.000
Guilt-Proneness	−0.25	−0.10	0.106	0.14	0.09	0.314	−0.51	−0.33	0.000	−0.07	−0.03	0.557
**Offender’s Relationship with the Sacred**												
Similarity of Offender Spirituality	−0.16	−0.17	0.005	0.25	0.46	0.000	−0.02	−0.04	0.625	−0.19	−0.24	0.000
**Relationship of the Transgression to the Sacred**												
Sacred Desecration	0.04	0.04	0.612	0.18	0.35	0.007	−0.06	−0.11	0.362	0.07	0.09	0.253

*Pre-training assessment of respective construct. T1 = respective outcome measured at Time 1.*

## Discussion

The present study largely supports the first hypothesis that forgiveness training will result in changes in forgiveness, emotional reactions, and self-esteem. Additionally, the present study provides the first evidence of the efficacy of the REACH Forgiveness program in India. This supports findings from a number of studies demonstrating the efficacy of this psychoeducational approach. For instance, the present findings are similar to a randomized controlled trial (RCT) of the REACH Forgiveness program that utilized undergraduate students (*N* = 97) in a 6-hour, in-person design. The REACH Forgiveness group showed greater improvements, compared to controls, in forgiveness at both post-test and 6-week follow-up assessments (Sandage and Worthington, 2010). Also, our results are similar to a study of 145 married couples randomly assigned to a control condition or a 9-hour, in-person, REACH Forgiveness counseling intervention with individual couples. Those in the REACH Forgiveness intervention reported greater improvements, compared to controls, in forgiveness, as well as, relationship quality, empathy, and negative mood across 1-, 3-, 6-, and 12-month follow-up assessments (Worthington et al., 2015). Again, similar to the present findings, a recent RCT with 162 middle-aged adults comparing a 12-hour, in-person, REACH Forgiveness group condition to a 12-hour, in-person, process group therapy condition and a wait-list control showed that the REACH Forgiveness group condition was better than the wait-list condition at reducing unforgiveness and rumination and increasing empathy and benevolence at mid-, and post-intervention and 6-month follow-up assessments, but REACH Forgiveness was equally effective as process group therapy focused on forgiveness on these same outcomes (Wade et al., 2018). Furthermore, several other studies have evaluated the REACH Forgiveness program using 6 to 13 h in-person trainings, downloadable workbooks, and online materials. Although the mode of delivery differs, the present psychoeducational groups show findings similar to samples from Australia, Ghana, and Indonesia and mixed foreign students studying in the United States (Worthington et al., 2010; Lin, 2012; Nation et al., 2018; Kurniati et al., 2020; Osei-Tutu et al., 2020).

Although the REACH Forgiveness program has been tailored and been shown to be effective with Christians (Lampton et al., 2005; Stratton et al., 2008; Worthington et al., 2010; Greer et al., 2014), the present study also offers the first evidence that a secular REACH Forgiveness program is effective in a largely Hindu sample (with a substantial minority of Muslims). It is important to note that the REACH Forgiveness program in the present study was not tailored to the Hindu, Muslim, or indeed any faith. Rather, the secular version of the program was implemented and yielded good benefits for the participants in mixed-religious groups. This outcome is reminiscent of the work of Rye et al. (2005) who evaluated the effectiveness of two versions of an eight-session forgiveness group intervention for divorced Christians. They described this intervention as “based loosely on Worthington’s (1998) REACH model of forgiveness” (p. 883). Participants (*N* = 149) were randomly assigned to a secular or a religiously accommodated (Christian) forgiveness condition, or to a no-intervention comparison condition. People in both intervention conditions reported more forgiveness of an ex-spouse than those in the control, but did not differ from each other. *Post hoc* interviews showed that Christians in both secular and religious interventions actually used the same religious coping strategies. Thus, the secular treatment worked as well as the religiously accommodated one to produce the focal outcome—forgiveness. This is common in psychotherapy intervention research on secular and religiously accommodated treatments. Captari et al. (2018), in a comprehensive recent meta-analysis of almost 100 religiously accommodated treatments, showed that secular and religiously accommodated treatments did not differ on the focal outcome. However, religiously-tailored interventions are typically more favorably received by religious participants and yield stronger spiritual benefits (Captari et al., 2018). Consequently, it might be worthwhile to invest in tailoring the REACH Forgiveness group program to suit the specific needs of the Hindu faith. Future research could determine whether this might yield even stronger forgiveness and emotional well-being benefits or more spiritual benefits.

Recall that Wade et al. (2014) characterized the dose-response relationship between effect sizes for forgiveness outcomes and amount of time spent in forgiveness training as: 0.10 + 0.05 (hours of intervention). Hence, we can derive an expected amount of gain in forgiveness given that participants in the present study were engaged in a 6-hour psychoeducational program. The predicted amount of change in forgiveness should be 0.4 standardized units of change. Immediate-training (*d*s ≥ 1.20) and wait-list comparisons (*d*s ≥ 0.65) both showed changes that notably exceeded this expected change. Wade et al. (2014) also reported aggregate changes in outcomes reflecting positive (i.e., hope) and negative affect (i.e., depression) as 1.00 and 0.34, respectively, which the present study effect sizes (*d*s ≥ 1.41 positive affect and *d*s ≥ 1.42 negative affect) again compare favorably too. Finally, Wade et al. (2014) showed that between-condition differences in forgiveness yielded standardized effect sizes of about 0.5, and in the present study between-condition differences at T2 were about twice that size (*d*s ≥ 1.02).

Given the impact of the REACH Forgiveness program in India, it is worthwhile considering what may have contributed to these powerful changes. First, the leader was an experienced and skilled facilitator who was trained by two of the co-authors (EW and SK). Hence, the fidelity of the training program, although not objectively measured, was likely quite high. Second, inclusion criteria for the sample was the experience of an event that remained, at minimum, substantially unforgiven and participants indeed reported severe hurt. Another inclusion criterion was that participants needed to be motivated to work on their unforgiveness in a group setting. These inclusion criteria likely created an environment in which participants shared and worked on significant past offenses and were motivated to get past them. Third, as a result of recent national events in India, forgiveness may be salient and there may presently be a favorable cultural climate for encouraging forgiveness (Mission India, 2017; Chandrasekharan, 2018). But, this may be fragile and short-lived, and current tensions between India and Pakistan as well as new governmental policies that marginalize Muslims highlight the tenuous nature of forgiveness, reconciliation, and peace in that region (BBC News, 2019; New York Times, 2019). Nevertheless, the participants in the present study appear to have been highly motivated to pursue forgiveness in their own personal lives and cultural factors are likely to influence these individual motivations.

The second hypothesis in this study was that relational spirituality variables would predict responsiveness to the intervention. For a few variables, this was true. However, for others it was not. Hence, hypothesis two was partially supported. Similarity of offender spirituality had the most consistent relationship to outcomes. Clearly consistent with the relational spirituality and forgiveness model (Davis et al., 2008; Worthington and Sandage, 2016), perceiving the offender as having a similar spirituality was related to less growth of unforgiveness and more growth in empathy, positive affect, and emotional forgiveness (though the similar spirituality and emotional forgiveness association only approached significance).

Other findings are less clearly explained by the relational spirituality and forgiveness model. For instance, perceived similarity of offender spirituality was related to less emotional expressivity and self-esteem—not more, as had been hypothesized. Shame-proneness was positively related to growth in emotional forgiveness, emotional expressivity, and self-esteem. Gratitude was negatively associated with growth in self-esteem. Guilt-proneness was negatively associated with growth in negative affect, and perceiving the offense as a sacred desecration was positively associated with growth in positive affect. When people perceive that a sacred desecration has occurred, they typically react with strong negative emotion (Pargament et al., 2005). Thus, one potential reason for this latter finding is merely that the initial reactions were so negative emotionally that regression to the mean accounted for the positive correlation. In the present study, we used the forgiveness and relational spirituality model for the first time to predict responsiveness to a forgiveness intervention rather than naturally occurring forgiveness. It could well be that different variables altogether are active. Additional research is needed on this and other interventions in which forgiveness and relational spirituality variables are examined.

### Limitations

Despite the considerable benefits that REACH Forgiveness training offered to participants in the present study, there are some limitations that should be mentioned. First, this is not a representative sample of Indians. Participants were all students of Karnatak University, all had considerable levels of education and were engaged in higher educational pursuits. These are characteristics that are not shared widely by all citizens of India. Second, although the effects of REACH Forgiveness training were of good size, all effects were measured through self-reported outcomes. The impact of REACH Forgiveness training on actual behavior, long-term attitudes, or visceral responses to an offender were not measured. Third, like most interventions, the internal validity of the study was high, but external validity may not be high. This psychoeducation took place in a highly controlled setting at a university psychology department. Fourth, overall the reliability of the measures in the present study was exceptional. In Table 1, we see that in 24 administrations of core, emotional, and self-esteem measures and five single-administration religious variables, only four administrations (of the 29) resulted in an alpha less than 0.7. One possible reason is that we pre-tested the questionnaires. In the instance of an exceptionally low reliability coefficient we urge caution in interpretation. This would be decisional forgiveness at T3. Three other marginal reliabilities were also observed for T2 decisional forgiveness (α = 0.63), T1 self-esteem (α = 0.56) and guilt-proneness (α = 0.67). However, in these latter cases the data certainly contain more measurement error but are still quite interpretable. This was not intended as a psychometric study, so it is not possible to determine the cause of these low or marginal estimated reliabilities. It is a point of concern because low estimated reliability increases measurement error which reduces the ability of the variable to correlate with other variables and lowers the sensitivity of the variable to experimental manipulations. Furthermore, the pre-testing phase of our work should not be interpreted as a cross-cultural validation of these measures. Future psychometric work should be done on these assessments.

## Conclusion

This is the first examination of the REACH Forgiveness program in India. The present study offers a strong and consistent pattern of findings in the immediate-training group that replicated in the wait-list comparison group and suggests REACH Forgiveness training is of utility in India. Findings from this study revealed benefits in forgiveness and unforgiveness, positive and negative affect, and spirituality outcomes, and all effect sizes were well greater than expected. Keeping in mind sample, self-report, and measurement limitations of the study, it appears that the evidence weighs strongly in favor of continued use and evaluation of the REACH Forgiveness program in India. No special adaptations were necessary to the program, a mostly Hindu sample responded very favorably, and future investigators may wish to determine what key features of program tailoring (e.g., religious) might offer additional improvements and reach. As the only empirically-tested forgiveness psychoeducation program in India at present, continued efforts to understand, enhance, and expand on REACH Forgiveness training in India would seem well-advised. The present study offers a step forward to REACH Forgiveness in one of the most divided and largest populations on earth.

## Data Availability Statement

The datasets generated for this study are available on request to the corresponding author.

## Ethics Statement

The study involving human participants was reviewed and approved by the Institutional Review Board at Karnatak University Dharwad. The participants provided their written informed consent to participate in this study.

## Author Contributions

All authors designed the study and shaped the hypotheses. EW and SK assisted in providing and organizing materials for IRB approval (i.e., training manuals, summary descriptions of the training, copies of measures) many of which were incorporated into the writing of the Materials and Methods. SK and SM oversaw all procedural aspects of the study. SM delivered the psychoeducational content, collected and entered data. LT conducted the analyses. LT, EW, AC, SK, and AB contributed to interpreting the results and to conceptualizing and assisting with editing the manuscript into its final form.

## Conflict of Interest

The authors declare that the research was conducted in the absence of any commercial or financial relationships that could be construed as a potential conflict of interest.

## References

[B1] Akhtar S. Barlow J. (2018). Forgiveness therapy for the promotion of mental well-being: a systematic review and meta-analysis. *Trauma Viol. Abuse* 19 107–122. 10.1177/1524838016637079 27009829

[B2] Batson C. D. Fultz J. Schoenrade P. A. (1987). Distress and empathy: two qualitatively distinct vicarious emotions with different motivational consequences. *J. Pers.* 55 19–39. 10.1111/j.1467-6494.1987.tb00426.x 3572705

[B3] BBC News (2019). *Kashmir: Why India and Pakistan Fight Over It.* London: BBC News.

[B4] Captari L. E. Hook J. N. Hoyt W. T. Davis D. E. McElroy S. E. Worthington E. L. Jr. (2018). Integrating clients’ religion and spirituality within psychotherapy: a comprehensive meta-analysis. *J. Clin. Psychol. Session* 74 1938–1951. 10.1002/jclp.22681 30221353

[B5] Chandrasekharan G. (2018). *Jessica Lall’s Sister, Rahul Gandhi Throw Some Light On The Power Of Forgiveness.* Available online at: https://www.mid-day.com/articles/the-power-of-maafi/19370172 (accessed September 2, 2019).

[B6] Davis D. E. Hook J. N. Worthington E. L. J. (2008). Relational spirituality and forgiveness: the roles of attachment to god, religious coping, and viewing the transgression as a desecration. *J. Psychol. Christ.* 27 293–301.

[B7] Davis D. E. Worthington E. L. Jr. Hook J. N. Van Tongeren D. R. Green J. D. Jennings I. (2009). Relational spirituality and the development of the Similarity of the Offender’s Spirituality Scale. *Psychol. Relig. Spirit.* 1 249–262. 10.1037/a0017581

[B8] Enright R. D. (2001). *Forgiveness is a Choice: A Step-By-Step Process For Resolving Anger And Restoring Hope.* Washington, DC: American Psychological Association.

[B9] Exline J. J. Worthington E. L. Jr. Hill P. McCullough M. E. (2003). Forgiveness and justice: a research agenda for social and personality psychology. *Pers. Soc. Psychol. Rev.* 7 337–348. 10.1207/S15327957PSPR0704_06 14633470

[B10] Fontaine J. R. Luyten P. De Boeck P. Corveleyn J. (2001). The test of self-conscious affect: internal structure, differential scales and relationships with long-term affects. *Eur. J. Pers.* 15 449–463. 10.1002/per.428

[B11] Greer C. L. Worthington E. L. Jr. Lin Y. Lavelock C. R. Griffin B. J. (2014). Efficacy of a self-directed forgiveness workbook for Christian victims of within-congregation offenders. *Spirit. Clin. Pract.* 1 218–230. 10.1037/scp0000012

[B12] Gross J. J. John O. P. (1997). Revealing feelings: facets of emotional expressivity in self-reports, peer ratings, and behavior. *J. Pers. Soc. Psychol.* 72 435–448. 10.1037/0022-3514.72.2.435 9107009

[B13] Gross J. J. John O. P. (2003). Individual differences in two emotion regulation processes: implications for affect, relationships, and well-being. *J. Pers. Soc. Psychol.* 85 348–362. 10.1037/0022-3514.85.2.348 12916575

[B14] Harper Q. Worthington E. L. Jr. Griffin B. J. Lavelock C. R. Hook J. N. Vrana S. R. (2014). Efficacy of a workbook to promote forgiveness: a randomized controlled trial with university students. *J. Clin. Psychol.* 70 1158–1169. 10.1002/jclp.22079 24619957

[B15] India Population (2019). Available online at: http://worldpopulationreview.com/countries/india/ (accessed August 31, 2019).

[B16] Kurniati N. M. T. Dwiwardani C. Worthington E. L. Jr. Widyarini N. Citra A. F. Widhiarso W. (2020). Does forgiving in a collectivistic culture affect only decisions to forgive and not emotions? REACH forgiveness collectivistic in Indonesia. *Intern. J. Psychol.* 10.1002/ijop.12648 31898323

[B17] Lampton C. Oliver G. J. Worthington E. L. Jr. Berry J. W. (2005). Helping Christian college students become more forgiving: an intervention study to promote forgiveness as part of a program to shape Christian character. *J. Psychol. Theol.* 33 278–290. 10.1177/009164710503300404

[B18] Lavelock C. R. Worthington E. L. Jr. Griffin B. J. Garthe R. C. Elnasseh A. Davis D. E. (2017). Still waters run deep: humility as a master virtue. *J. Psychol. Theol.* 45 286–303. 10.1177/009164711704500404

[B19] Lin Y. (2012). *Efficacy of Reach Forgiveness for Foreign and Virginia Students.* Unpublished Master’s thesis, Virginia Commonwealth University, Richmond, VA.

[B20] Lin Y. Worthington E. L. Jr. Griffin B. J. Greer C. L. Opare-Henaku A. Lavelock C. R. (2014). Efficacy of REACH forgiveness across cultures. *J. Clin. Psychol.* 70 781–793. 10.1002/jclp.22073 24493237

[B21] Luskin F. (2002). *Forgive for Good: A Proven Prescription for Health and Happiness.* New York, NY: HarperCollins.

[B22] McCullough M. E. Bellah C. G. Kilpatrick S. D. Johnson J. L. (2001). Vengefulness: relationships with forgiveness, rumination, well-being, and the big five. *Pers. Soc. Psychol. Bull.* 27 601–610. 10.1177/0146167201275008

[B23] McCullough M. E. Emmons R. A. Tsang J.-A. (2002). The grateful disposition: a conceptual and empirical topography. *J. Per. Soc. Psychol.* 82 112–127. 10.1037/0022-3514.82.1.112 11811629

[B24] McCullough M. E. Luna L. R. Berry J. W. Tabak B. A. Bono G. (2010). On the form and function of forgiving: modeling the time-forgiveness relationship and testing the valuable relationships hypothesis. *Emotion* 10 358–376. 10.1037/a0019349 20515225

[B25] McCullough M. E. Rachal K. C. Sandage S. J. Worthington E. L. Jr. Brown S. W. (1998). Interpersonal forgiving in close relationships: II. Theoretical elaboration and measurement. *J. Pers. Soc. Psychol.* 75 1586–1603. 10.1037/0022-3514.75.6.1586 9914668

[B26] McCullough M. E. Root L. M. Cohen A. D. (2006). Writing about the benefits of an interpersonal transgression facilitates forgiveness. *J. Consul. Clin. Psychol.* 74 887–897. 10.1037/0022-006X.74.5.887 17032093

[B27] McCullough M. E. Worthington E. L. J. Rachal K. C. (1997). Interpersonal forgiving in close relationships. *J. Pers. Soc. Psychol.* 73 321–336. 10.1037/0022-3514.73.2.321 9248052

[B28] Mission India (2017). *The Power Of Forgiving.* Available online at: https://missionindia.org/the-power-of-forgiving/ (accessed August 31, 2019).

[B29] Nation J. A. Wertheim E. H. Worthington E. L. Jr. (2018). Evaluation of an online self-help version of the reach forgiveness program: outcomes and predictors of persistence in a community sample. *J. Clin. Psychol.* 74 819–838. 10.1002/jclp.22557 29244197

[B30] New York Times (2019). *India Steps Toward Making Naturalization Harder For Muslims.* Available online at: https://www.nytimes.com/2019/12/09/world/asia/india-muslims-citizenship-narendra-modi.html (accessed January 12, 2020).

[B31] Niezink L. W. Siero F. W. Dijkstra P. Buunk A. P. Barelds D. P. H. (2012). Empathic concern: distinguishing between tenderness and sympathy. *Motiv. Emot.* 36 544–549. 10.1007/s11031-011-9276-z 23144514 PMC3491184

[B32] Osei-Tutu A. Osafo J. Anum A. Appiah-Danquah R. Worthington E. L. Jr. Nonterah C. W. (2020). Is cultural adaptation needed beyond using Christian-accommodated reach forgiveness psychoeducational group intervention in Ghana? An efficacy study comparing a Christian-accommodated version against a version accommodated by Christian and cultural adaptations. *Spirit. Clin. Pract.* 10.1037/scp0000215

[B33] Pargament K. I. Magyar G. M. Benore E. Mahoney A. (2005). Sacrilege: a study of sacred loss and desecration and their implications for health and well-being in a community sample. *J. Sci. Study Relig.* 44 59–78. 10.1111/j.1468-5906.2005.00265.x

[B34] Rosenberg M. (1965). *Society and the Adolescent Self-Image.* Princeton, NJ: Princeton University Press.

[B35] Rye M. S. Pargament K. I. Ali M. A. Beck G. L. Dorff E. N. Hallisey C. (2000). “Religious perspectives on forgiveness,” in *Forgiveness: Theory, Research, and Practice*, eds McCullough M. E. Pargament K. I. Thoresen C. E. (New York, NY: Guilford Press), 17–40.

[B36] Rye M. S. Pargament K. I. Pan W. Yingling D. W. Shogren K. A. Ito M. (2005). Can group interventions facilitate forgiveness of an ex-spouse? A randomized clinical trial. *J. Consul. Clin. Psychol.* 73 880–892. 10.1037/0022-006x.73.5.880 16287388

[B37] Sandage S. J. Long B. Moen R. Jankowski P. J. Worthington E. L. Jr. Wade N. G. (2015). Forgiveness in the treatment of borderline personality disorder: a quasi-experimental study. *J. Clin. Psychol.* 71 625–640. 10.1002/jclp.22185 25877954

[B38] Sandage S. J. Worthington E. L. Jr. (2010). Comparison of two group interventions to promote forgiveness: empathy as a mediator of change. *J. Ment. Health Counsel.* 32 35–57. 10.17744/mehc.32.1.274536n518571683

[B39] Sinclair S. J. Blais M. A. Gansler D. A. Sandberg E. Bistis K. LoCicero A. (2010). Psychometric properties of the rosenberg self-esteem scale: overall and across demographic groups living within the United States. *Eval. Health Prof.* 33 56–80. 10.1177/0163278709356187 20164106

[B40] Stratton S. P. Dean J. B. Nonneman A. J. Bode R. A. Worthington E. L. Jr. (2008). Forgiveness interventions as spiritual development strategies: comparing forgiveness workshop training, expressive writing about forgiveness, and retested controls. *J. Psychol. Christ.* 27 347–357.

[B41] Tangney J. P. (1990). Assessing individual differences in proneness to shame and guilt: development of the self-conscious affect and attribution inventory. *J. Pers. Soc. Psychol.* 59 102–111. 10.1037/0022-3514.59.1.102 2213483

[B42] Tangney J. P. (1996). Conceptual and methodological issues in the assessment of shame and guilt. *Behav. Res. Therapy* 34 741–754. 10.1016/0005-7967(96)00034-4 8936757

[B43] Tangney J. P. Dearing R. L. Wagner P. E. Gramzow R. (2000). *The Test of Self-Conscious Affect Version 3 (TOSCA-3).* Fairfax, VA: George Mason University.

[B44] Toussaint L. Worthington E. L. Jr. Williams D. R. (2015). *Forgiveness and Health: Scientific Evidence And Theories Relating Forgiveness To Better Health.* New York, NY: Springer.

[B45] Wade N. G. Cornish M. A. Tucker J. R. Worthington E. L. Jr. Sandage S. J. Rye M. S. (2018). Promoting forgiveness: characteristics of the treatment, the clients, and their interaction. *J. Couns. Psychol.* 65 358–371. 10.1037/cou0000260 29672085

[B46] Wade N. G. Hoyt W. T. Kidwell J. E. M. Worthington E. L. (2014). Efficacy of psychotherapeutic interventions to promote forgiveness: a meta-analysis. *J. Consul. Clin. Psychol.* 82 154–170. 10.1037/a0035268 24364794

[B47] Watson D. Clark L. A. Tellegen A. (1988). Development and validation of brief measures of positive and negative affect: the PANAS scales. *J. Pers. Soc. Psychol.* 54 1063–1070. 10.1037//0022-3514.54.6.1063 3397865

[B48] Worthington E. L. Jr. (1998). “The pyramid model of forgiveness: some interdisciplinary speculations about unforgiveness and the promotion of forgiveness,” in *Dimensions of Forgiveness: Psychological Research & Theological Perspectives*, ed. Worthington E. L. Jr. (Radnor, PA: Templeton Foundation Press), 107–137.

[B49] Worthington E. L. Jr. (2008). *Steps to Reach Forgiveness And To Reconcile.* Upper Saddle River, NJ: Pearson.

[B50] Worthington E. L. Jr. (2020). “An update of the REACH forgiveness model to promote forgiveness,” in *Handbook of Forgiveness*, 2nd Edn, eds Worthington E. L. Jr. Nathaniel G. Wade (New York, NY: Routledge), 277–287. 10.4324/9781351123341-26

[B51] Worthington E. L. Jr. Berry J. W. Hook J. N. Davis D. E. Scherer M. Griffin B. J. (2015). Forgiveness-reconciliation and communication-conflict-resolution interventions versus retested controls in early married couples. *J. Couns. Psychol.* 62 14–27. 10.1037/cou0000045 25264599 PMC6280976

[B52] Worthington E. L. Jr. Hook J. N. Utsey S. O. Williams J. K. Neil R. L. (2007). “Decisional and emotional forgiveness,” in *Paper Presented at the International Positive Psychology Summit*, Washington, DC.

[B53] Worthington E. L. Jr. Hunter J. L. Sharp C. B. Hook J. N. Van Tongeren D. R. Davis D. E. (2010). A psychoeducational intervention to promote forgiveness in Christians in the Philippines. *J. Ment. Health Couns.* 32 75–93. 10.17744/mehc.32.1.t3677072811lj864

[B54] Worthington E. L. Jr. Sandage S. J. (2016). *Forgiveness and Spirituality in Psychotherapy: A Relational Approach.* Washington, DC: American Psychological Association.

[B55] Worthington E. L. Jr. Sandage S. J. Berry J. W. (2000). “Group interventions to promote forgiveness: What researchers and clinicians ought to know,” in *Forgiveness: Theory Research and Practice*, eds McCullough M. E. Pargament K. I. Thoresen C. E. (New York, NY: Guilford Press), 228–253.

[B56] Worthington E. L. Jr. Sandage S. J. Ripley J. S. (2018). “Strategies to facilitate forgiveness: REACH Forgiveness model,” in *Counseling techniques: A Comprehensive Resource for Christian Counselors*, eds John Thomas C. (Grand Rapids, MI: Zondervan), 417–437.

